# Designing a ‘Deep End’ childhood immunisation intervention: a pre-implementation qualitative study

**DOI:** 10.3399/BJGPO.2025.0110

**Published:** 2026-04-22

**Authors:** Amber Sacre, Adam Todd, Clare Bambra, Sarah Sowden

**Affiliations:** 1 Population Health Sciences Institute, Newcastle University, Newcastle-upon-Tyne, UK; 2 National Institute for Health and Care Research (NIHR) Applied Research Collaboration (ARC) North East and North Cumbria (NENC), Newcastle-upon-Tyne, UK; 3 School of Pharmacy, Newcastle University, Newcastle-upon-Tyne, UK; 4 Newcastle Patient Safety Research Collaboration (PSRC), Newcastle University, Newcastle-upon-Tyne, UK

**Keywords:** health inequalities, vaccine uptake, healthcare intervention, vaccination

## Abstract

**Background:**

Childhood vaccination uptake is declining in England. Efforts are required to prevent further decline and safeguard against outbreaks of vaccine-preventable diseases (VPDs). Individuals in areas of high socioeconomic disadvantage can face greater barriers to vaccination and, subsequently, be at an increased risk of lower uptake and VPDs.

**Aim:**

To conduct a pre-implementation evaluation of a proposed childhood ‘catch-up’ immunisation pilot intervention targeted at North East and North Cumbria ‘Deep End’ (NENC DE) Network general practices.

**Design & setting:**

The NENC DE is a network of general practices serving populations experiencing high levels of socioeconomic disadvantage. The proposed intervention would provide additional staffing capacity (one administrator and two vaccination-trained nurses) to be used discretionally by each NENC DE practice; tasks could include offering an in-home roving vaccination service or identifying children not vaccinated to schedule.

**Method:**

Semi-structured qualitative interviews were employed, and a purposive and snowball sampling approach was adopted. Fifteen interviews were conducted in 2023 - the initial commissioning phase of the intervention. Transcripts were analysed using the framework method.

**Results:**

The proposed intervention was received with intrigue, especially the roving service, which would reduce some practical access barriers for families. Success could be measured by an increase in childhood vaccination uptake, hopefully among the most vulnerable children. However, concerns regarding physical space and staff safety were raised.

**Conclusion:**

We encourage the NENC DE intervention providers to ensure the service remains flexible and customisable. Further research will be required to assess its impact and effectiveness when underway.

## How this fits in

Childhood vaccination uptake is declining in England,^1,2^ and experts in the field are urgently calling for efforts to prevent further decline and protect children from vaccine-preventable diseases.^3^ We conducted a pre-implementation evaluation of a targeted, childhood ‘catch-up’ immunisation pilot intervention in ‘Deep End’ general practices in the North of England serving populations experiencing high levels of socioeconomic disadvantage. We explore how an at-home vaccination roving service may be best suited for increasing uptake among vulnerable children and those from families who face greater barriers to accessing vaccination services. This is especially relevant in light of the NHS 10-Year Health Plan for England, which promises new models of the health visitor service to increase vaccination coverage in disadvantaged groups.^4^

## Introduction

In England, none of the 14 childhood vaccinations administered before the age of 5 years^5^ achieved the 95% coverage target as outlined for the 2023–2024 evaluation period.^6^ This is reflective of longer-term national trends, demonstrating a gradual decline over the past decade, with an increased rate of decline during the COVID-19 pandemic nationally and globally.^2,7^ Low vaccination coverage leads to an increase in vaccine-preventable disease outbreaks, as was the case with measles in Birmingham and London over recent years.^8^

One group at an increased risk of poor childhood health in general, and lower childhood vaccination uptake, are children living in areas of higher socioeconomic disadvantage.^9,10^ Research links this lower uptake to a potentially greater prevalence of barriers to uptake, such as childcare considerations and complex personal lives that take priority over vaccination appointment attendance.^10–14^ The North East of England has the highest coverage for most childhood vaccines compared with any other region.^2^ However, there is still considerable variability at the general practice level; some practices serving socioeconomically disadvantaged communities experience lower uptake than the regional average.^6^ Research into effective interventions to improve childhood vaccination uptake suggests complex, targeted approaches designed with a specific population in mind are most successful.^15–17^

This study explores the opinions of stakeholders involved in the development of a childhood vaccination intervention commissioned by the North East and North Cumbria ‘Deep End’ (NENC DE) Network before implementation. The Deep End general practice networks originated in Scotland in 2009, and were created to support GPs nationally and internationally^11^ who work in areas of high socioeconomic disadvantage through various pathways, including policy advocacy, commissioning, and supporting interventions.^18^ They were formed in recognition that patients living in these areas often have higher morbidity and mortality, multiple complex needs, and lower levels of engagement in preventive healthcare services (such as immunisation), but (comparatively) greater use of urgent and emergency care than patients living in less socioeconomically disadvantaged areas.^18^ On average, Deep End healthcare professionals care for more patients per GP, contributing to increased stress levels and burnout.^18,19^ These contexts often make it difficult to recruit and retain healthcare workers^20^ and, consequently, result in lower overall staffing levels per patient.^21^

The NENC DE was established in 2020. In 2023, when the data collection for this study took place, the network comprised 38 general practices. Co-design work between NHS England Public Health Programmes Team and professionals working in the Deep End uncovered frustration with performance-driven targets,^22^ namely the Quality Outcome Framework (QOF),^23^ in areas such as immunisation. These initiatives were perceived as unfairly financially penalising practices in areas of greater socioeconomic disadvantage and as reflective of a broader healthcare system that failed to appreciate the challenges and complexities of practising in this context.^11^ GPs serving these communities require additional support.^11^

In response to the discussed challenges of practising in the NENC DE and the low childhood vaccination uptake rates identified in some member practices, an immunisation ‘catch-up’ intervention was conceptualised. The proposed opt-in intervention would involve two multidisciplinary teams comprised of one administrator and two vaccination-trained nurses. Each team would undertake a 2-week rotation in each interested NENC DE practice, offered in order of need and beginning with those with the lowest uptake levels. Table 1 provides an overview of the intervention.

**Table 1.** Details of the proposed North East and North Cumbria Deep End Network childhood immunisation “catch-up” pilot intervention

Aim
To increase timely childhood vaccination uptake according to schedule and ensure the integrity of immunisation records.
**Proposed Specification**
**Staffing:** Two vaccination-trained nurses and one administrator, employed by a vaccination service delivery partner organisation. **Duration:** Two-week rotation in each interested North East and North Cumbria Deep End (NENC DE) practice. **Rollout procedure:** Offered to NENC DE practices in order of need, beginning with those with the lowest childhood vaccination coverage.
**Suggested Tasks**
The proposed service was designed to provide flexible, extra staffing capacity. As such, each NENC DE practice could exercise their discretion regarding the specific tasks undertaken. However, some examples of these tasks are outlined as follows: **…Administrator** Identifying and verifying children not vaccinated to schedule, Contacting parents to arrange vaccination appointments, Ensuring consistency between general practices and Child Health Information Services (CHIS) records of children not vaccinated to schedule. **…Nurses** Delivering additional/extended hours vaccination clinics in the general practice to children not vaccinated to schedule, Offering a roving service to vaccinate children not vaccinated to schedule in their homes, if likely to be more successful in removing or minimising access barriers than attending the practice, Providing a “family offer”, where siblings can be vaccinated together in one appointment, Recording vaccination uptake accurately and timely after occurrence.

The primary objective of the proposed intervention was to provide a flexible service that each practice could tailor to its needs. Owing to the high degree of flexibility required, the ‘catch-up’ pilot is classed as a complex intervention.^24^ It was hoped the intervention would increase timely vaccination uptake and reduce the burden of delivering the programme through additional staffing. The involvement of research in the development, pre-implementation, implementation, and final evaluation of interventions is crucial to ensure there is an evidential impact on the challenges it is addressing.^24^ In the context of nationally and globally declining vaccination uptake,^6,7^ the findings of this study can be applied to other contexts seeking to improve their vaccination coverage, specifically in areas of high socioeconomic disadvantage, including other Deep End networks.^11^ Therefore, we conducted a pre-implementation evaluation of a proposed childhood ‘catch-up’ immunisation pilot intervention targeted at NENC DE general practices.

## Method

Semi-structured interviews were conducted by a member of the NENC DE research team (AS) with professionals involved (in varying capacities) in the pilot intervention. Specifically, those who: (1) worked in NENC DE member practices (nurses, GPs, practice or operations or business managers), including NENC DE management; (2) supported, monitored, or commissioned the childhood vaccination programme in the North East of England. Any staff member working in an NENC DE practice was eligible if they had a connection to the childhood vaccination programme. However, those from the wider vaccination system must have *overseen* a childhood vaccination-related work package and be aware of the proposed NENC DE ‘catch-up’ intervention; eligibility was assessed on a case-by-case basis. Interviews were scheduled to take place in 2023 during the initial commissioning phase of the service. At this point, the intervention was not yet operational.

The NENC DE email mailing list, a database of all members’ contact information, was utilised to facilitate recruitment. Additionally, the NENC DE hosts many in-person networking events where the pilot intervention and the pre-implementation study were promoted. Thus, purposive sampling could be undertaken with member practices who had expressed an interest in the intervention. Through NENC DE connections, those working in regional NHS England commissioning teams and local authority public health teams in the North East were invited to participate. Contact details of other eligible professionals were requested from those who declined participation, and at the end of each interview (snowball sampling). A Participant Information Sheet detailing the study’s particulars was provided, and a completed electronic consent form was requested to confirm an individual’s informed decision to participate in the interview. Ten NENC DE employees were sought for participation, and 10 from the wider childhood vaccination system (20 total). However, recruitment would cease if *a priori* thematic saturation was attained before this amount was achieved.^25^

To avoid occupying more participants’ time than necessary, all interviews were conducted online using Zoom or Microsoft Teams and were audio-video recorded using the built-in facilities. The interview schedule (Supplementary material S1) was planned to last a maximum of 1 hour, and covered topics such as overall opinions of the intervention, what ‘success’ would look like, and foreseeable challenges of implementation. For local authority and regional-level participants, the topics were first discussed in general and then with reference to areas of high socioeconomic disadvantage. The questions were phrased as relevant to the professional role of participants. Where appropriate, prompts and follow-up questions were used to probe further into responses.

The interviews were transcribed verbatim by the research team. Names and other identifying information were removed, and the transcripts were labelled according to job role. NVivo (version 14)^26^ was used to facilitate the analysis, which took a framework approach developed for large-scale policy research.^27^ A framework approach allowed for the accounts of different professionals to be easily identifiable in the analysis (for more information, see Supplementary material S2). An independent researcher (TP) acted as a secondary rater to verify that the processes of coding and theme generation were carried out in a systematic and credible manner. A completed consolidated criteria for reporting qualitative research (COREQ) checklist^28^ is presented in Supplementary Material S3.

## Results

Fifteen interviews were conducted between June 2023 and December 2023; the details of which are presented in Table 2. The response rate was low, and several potential participants declined involvement, citing staffing shortages. We determined that *a priori* saturation was achieved after 13 interviews, when a diversity of opinions on the intervention pilot had been established, and the codes generated were increasingly similar. Two further key stakeholders in the childhood vaccination programme were identified and sought for participation, as they would have important reflections on the policy implications of the pilot. Subsequently, no further interviews were conducted after the 15 were concluded; at this stage, we had sufficient information to provide feedback to the prospective intervention providers, informing further development.

**Table 2.** Interview participant details (*n* = 15)

Participant details	Geographical location	Occupation details
**Deep End Network NENC member practice professionals** (*n* = 7) (*deliver the childhood vaccination programme*)		
Nurse 1	Newcastle	Nurse practitioner
Nurse 2	Newcastle	
GP 1	Sunderland	GP and practice partner
GP 2	Gateshead	
GP 3	Sunderland	
Practice manager 1	Middlesborough	Practice manager
Practice manager 2	South Shields	
**Professionals involved in the wider childhood vaccination system** (*n* = 8) (*commission, support, and monitor the childhood vaccination programme*)		
GP 4	Durham	GP and practice partner
Commissioner 1	North East and Yorkshire	NHS England North East and Yorkshire
Commissioner 2		
Commissioner 3		
Commissioner 4		
Healthcare delivery partner 1	North East and Yorkshire	A private organisation that supports healthcare delivery
Public health employee 1	Middlesborough	Local authority public health team
Public health employee 2	Hartlepool	

The following three themes were identified during the analysis: (1) intervention rationale and anticipated benefits; (2) issues of implementation; and (3) beyond the pilot.

### Intervention rationale and anticipated benefits

Theme 1 explored the rationale behind the intervention and its anticipated benefits, uncovering a range of differing viewpoints and perspectives across the professionals interviewed. The intervention was reportedly the product of two behavioural insights work packages conducted by commissioners in the NHS England Screening and Immunisations Team; the first with NENC DE members and the second with parents and stakeholders. In the first work package, NENC DE professionals identified several barriers to childhood vaccination for their patient population but felt limited in their ability to address them.

*‘… they* [NENC DE professionals involved in behavioural insights work] *had an extremely good understanding of why the communities couldn't engage, but there was a lot that was outside of what they could do.*’ (Commissioner 1)

One of the primary findings of the insights work was that, in some instances, low uptake in NENC DE practices was not necessarily related to vaccine-hesitant views but physical barriers to access.

*‘… they* [families registered at NENC DE general practices] *are not resistant to vaccination; they* [NENC DE general practices] *just can't get them through the doors for some reason.*’ (Commissioner 1)

Thus, the aim and focus of the proposed intervention was to use ordinary routes of engagement in an enhanced, flexible way by offering additional staffing capacity. This was seen as hopefully having a three-fold impact:

*‘… we* [would] *expect an increase in uptake, and therefore, that has a public health benefit to the whole of the population, not just the individual child, in terms of trying to reach herd immunity within a population ... there’s an individual child benefit, but then there is a system benefit that will enable those practices to reach their QOF targets and therefore release the income that is associated with that attainment ...*’ (Healthcare delivery partner 1)

Additionally, participants suggested that being able to vaccinate the most *‘vulnerable*’ children, such as those experiencing safeguarding issues, would demonstrate that the intervention had been successful. Interviewees defined safeguarding issues, for example, as families with child protection concerns (such as, abuse or neglect), involvement in the judicial system, or were looked-after children.

‘[Discussing potential benefits of the intervention] *I just think improving the uptake, particularly if we get* [vaccinate] *vulnerable children who haven't been immunised before* …’ (GP 2)

‘*…they're the families that we’re mainly concerned about, the ones who are on our list that we discuss at safeguarding meetings …*’ (GP 2)

Overall, providers viewed the proposed intervention with intrigue, albeit with some measured scepticism. The nurses interviewed were the most enthusiastic, reporting that the intervention would benefit their practices, but in different forms. One nurse felt that additional staffing capacity to offer a more diverse range of vaccination appointments would be helpful owing to their personal overwhelming workload.

*‘I think we probably would benefit … because I'm the only practice nurse, and my clinics are full all of the time for all appointments until they put extra appointments on in a month’s time …*’ (Nurse 2)

Nurse 2 also disclosed that their practice managers *‘didn’t agree*’ that additional staffing was required, leaving them feeling frustrated and burntout.

The potential for a roving element, where children are vaccinated in their homes, was most favourably received. This would remove some physical access barriers for families that find it difficult to attend the practice for vaccination appointments.

*‘… definitely trying to access people’s homes would help, because sometimes they're just really busy, and they've got different issues in their life that, for them, are more important than vaccinating their children. I think if somebody came to them and they didn't have to try and organise things and get them there. A lot of our families have multiple children, and it’s just difficult to try and organise, especially if they’re young, or if they’re in school …*’ (Nurse 2)

However, participants were less enthusiastic about the administrative support, indicating this may not be required for all practices and, subsequently, demonstrating the need for flexibility in the intervention model.

*‘*[Discussing administrative support] *… we’ve got a little system going, so we’re OK.*’ (Nurse 1)

It was suggested that preparatory work on behalf of the practice would be needed to maximise the effectiveness of the 2-week intervention period. This may require priorly identifying children not vaccinated to schedule and preparing general practice staff for the intervention team. Some participants were more receptive to the thought of implementing the required changes, but others were concerned that this would create additional work for the practice.

‘… *I’ll try anything that’s going to help me get better … I’m up for any changes, any improvements, absolutely.*’ (Practice manager 2)

*‘Some practices will be so stretched that they'll not want to do it. They'll expect the* [NENC DE] *Network to do everything. But I think if we do some of that stuff* [preparatory work] *first, the practices will get a better impact.’* (GP 2)

### Issues of implementation

The second theme to emerge was the potential issues of implementation. Both practical and implicit implementation concerns were reported. Practical concerns were, for example, clinical and non-clinical physical space for the intervention team to work and access IT systems.

*‘We could potentially struggle with space. All of our rooms are full all of the time, or a lot of the time.’* (Nurse 2)

‘*There might be technical issues about getting people* [the intervention team] *onto their clinical system in time to deliver to the intervention*.’ (GP 2)

Implicit concerns referred to the safety and reception of the intervention team. As evidenced, children experiencing safeguarding concerns are at risk of lower vaccination uptake, and considering this, one aim of the pilot was to vaccinate those most vulnerable. Subsequently, there was a concern that the roving team could enter potentially unsafe environments. This is a difficult concern to navigate. Children in these situations would benefit the most from a roving service, but staff safety is equally important.

*‘… making sure that there’s somewhere on the form that the practice submits to say, “Don’t go to these people”, for any violence or anything, because you need to exclude them. You don't want nurses walking into a house where patients could be potentially violent ...*’ (Practice manager 1)

Excluding these children from the intervention would be a significant oversight, and there can be many reasons for safeguarding concerns unrelated to violence. More investigation into this would be needed to ensure the safety of all parties.

Another implicit implementation issue was that families may be less receptive to the unfamiliar intervention team. One participant suggested the characteristics of the nurses selected for this role would be vital in ensuring its success; they would need to put families at ease despite their lack of familiarity, especially if they are vaccine-hesitant or have complex personal lives.

*‘… I suppose it’s who’s in that roving team. I think for me, being a health visitor who had a caseload, and a relationship with those parents, and an awareness of the communities and challenges they lived in, it was quite beneficial in being trusted.*’ (Public health employee 2)

### Beyond the pilot

The third theme that arose from the data was participants' views ‘beyond the pilot’, relating to the long-term impact of the intervention. One participant reported that they would welcome the intervention if the team were equipped with knowledge on how to improve vaccination uptake in disadvantaged communities. Learning new approaches to delivery and being supported by the NENC DE was equally important as a more practical, such as financial, means of support. This knowledge would remain with the practice beyond the 2-week intervention period.

‘*… if we could have a meeting beforehand with the team, a couple of weeks, or a few weeks before they come, so we understand what's worked elsewhere, particularly with communities I'm not familiar with …’* (GP 3)

*‘… it's not even so much funding, help and support is enough …’* (Practice manager 2)

From a commissioning perspective, the legacy of intervention was seen as problematic. Decision-makers responsible for high-level financial allocations reportedly struggle to accurately gauge the actual resources required to achieve current outcomes when multiple small-scale initiatives distort the overall picture.

*‘… the cost model is really what’s paid from the GP contract. And all this other activity that we're doing around the outside of it: is anybody doing the sums to put in that other activity, to get a clear view of really what is the financial cost of having these programmes?*’ (Commissioner 3)

Thus, if the intervention were to continue, investigations into the health-economic cost would be required. Interviewees reported that a thorough evaluation of the pilot would be crucial to this process.

*‘… if we can prove the impact, then why wouldn't we keep finding a way to do it* [continue the intervention] *for patients? That’s why the work and the evaluation and the research is so important …*’ (GP 2)

While the NENC DE childhood immunisation intervention is a new creation, vaccinating children in the home is not; health visitors used to offer this service. Local authorities commission health visitors, as part of the Healthy Child Programme for children and young people aged 0–19 years, which participants felt does not currently function properly.

‘*Our health visitor services is ... the word “disarray” may do it a disservice, but I think it’s similar in a lot of areas.*’ (GP 4)

One public health employee reported having previously worked as a health visitor when they used to vaccinate. They recalled the importance of the health visitor, not only for vaccinating but also for addressing vaccine hesitancy owing to the relationships built with families.

*‘…we* [health visitors] *would give them their first immunisations and follow the schedule through. The benefits of that approach is that you really had a really good rapport with parents, carers and children. And also, we would do opportunistic catch-ups, so we would do them in the home.*’ (Public health employee 2)

One commissioner involved in the insights work that informed the intervention (discussed in theme 1) explained that participants in their focus groups also recalled the usefulness of the health visitor service.

*‘*[imitating focus group participant from insights work] *…“it’s all very well having all these leaflets and going on the internet and everything but when I had kids” – lots of these were grandparents – “your health visitor came, and they really talked to you about the vaccines”…*’ (Commissioner 2)

Thus, there are already mechanisms to deliver this as a consistent service, but it would require reallocating funding and expanding the health visitor service.

*‘I think that as a system, as a whole, we may be not using that part* [health visitors] *well, particularly of that last 10–15% of parents who aren’t bringing their children along. And so, we could be making more use of the health visitors, but a part of that is making sure that that service is well specified and obviously funded to do that work. It takes time.’* (Commissioner 3)

## Discussion

### Summary

This study conducted a pre-implementation evaluation of a proposed childhood ‘catch-up’ immunisation pilot intervention targeted at NENC DE general practices. The intervention was received positively, especially the roving service, which would reduce access barriers for some families. It was hoped that there would be an increase in childhood vaccination coverage, particularly among those most vulnerable, and that this would subsequently enable general practices to receive their QOF payments. Flexibility in intervention delivery was of the utmost importance, as, despite their NENC DE commonality, general practices have unique requirements. For instance, some participants reported that an administrator was not needed. However, concerns were raised regarding physical space, IT system access, and staff safety. The importance of carefully selecting the roving team members was highlighted; they must be able to foster trust in a short period of time to encourage vaccination despite their unfamiliarity. The third theme further supported this notion, which discussed utilising and expanding existing mechanisms to build patient–provider trust and vaccinate children in their homes, such as the health visitor service.

### Strengths and limitations

A strength of our study is that we interviewed a diverse cross-section of professionals from a range of organisations across the childhood vaccination system, such as commissioners and local authority public health employees, to ensure the implications of the intervention were considered from both policy and practice perspectives. We provided a critical overview of a proposed childhood immunisation ‘catch-up’ intervention, demonstrating the strengths and weaknesses of the proposed model.

A limitation of our research was that we did not have the opportunity to interview any general practice professionals with the lowest levels of childhood vaccination uptake who would be prioritised in the intervention roll-out, whose insights would have been invaluable in informing its development. These practices were contacted to participate, but owing to the staffing pressures previously documented,^11,26^ they were unable to do so.

### Comparison with existing literature

A systematic review by Crocker-Burque *et al* ^27^ explored interventions to reduce inequalities in vaccine uptake among children and adolescents aged <19 years. The authors stated that *‘complex, locally designed interventions demonstrated the best evidence for effectiveness in reducing inequalities in deprived, urban, ethnically diverse communities*’.^27^ Our research is consistent with this finding, as the need for flexibility was required so practices can tailor the ‘catch-up’ pilot intervention to address their specific needs. This was also evident in other NENC DE studies.^29^ However, it must be acknowledged that offering services with significant flexibility can prove challenging to commission, implement, and manage.^24^

Our research extends current thinking in this area in relation to the suitability of in-practice vaccination models, specifically the strengths and challenges of designing a ‘roving’ service. Important to this study is the recognition that some families are not suited to the in-practice model of childhood vaccination delivery. Individuals with complex personal lives, of which there is a greater prevalence registered at Deep End general practices, require a different approach.^11^ Research recognises this and calls for a change in the narrative when discussing provision in areas of high socioeconomic disadvantage.^11,29^ During the COVID-19 pandemic, various approaches to vaccination delivery were employed to maximise uptake, including offering a range of convenient vaccination locations.^30,31^ We recognise that this is more challenging in the context of childhood vaccinations, especially those delivered at 8, 12, and 16 weeks, as these may be coupled with new-baby health checks. Nevertheless, there are lessons to be learnt from the pandemic regarding diversifying vaccination locations and decentralising provision.^30^

### Implications for research and practice

The providers of the NENC DE ‘catch-up’ intervention can utilise this study’s findings to shape the implemented model and enable them to consider issues that may not have been recognised. For example, the views shared indicate it may not be necessary to provide the administrator as standard, and instead, assess their appropriateness on a case-by-case basis. After these considerations are reflected on, families registered at NENC DE general practices should be engaged in patient and public involvement and engagement (PPIE) research to ensure the intervention appropriately addresses some of their barriers to uptake.

Second, the findings of this study can be applied to other areas seeking to improve their vaccination coverage, specifically in areas of high socioeconomic disadvantage and other Deep End networks;^11^ this is especially relevant in the context of nationally and globally declining vaccination coverage.^6,7^ For example, ensuring processes are in place to address potential safety concerns on behalf of the intervention team is an important universal consideration of organisations seeking to implement a roving service in any capacity. This is especially relevant in light of the NHS 10-Year Health Plan for England, which promises new models of the health visitor service to increase vaccination coverage in disadvantaged groups.^4^

Lastly, research at key stages of intervention development is key to ensuring they are *‘implementable, cost-effective, transferable, and scalable’.* ^24^ Further efficacy and cost-effectiveness evaluations of the ‘catch-up’ immunisation intervention are needed to assess its potential longer-term impact, which is especially relevant in light of the call for integrated care boards (ICBs) to reduce running costs by 50%.^32^ This intervention is one of several commissioned by the NENC DE (for example, supporting opioid deprescribing,^29^ workforce recruitment and retention,^20,33^ and mental health and multiple complex needs).^34^ Additional research to identify and synthesise common themes and intervention components would be beneficial to their future commissioning, specifically those targeted at socioeconomically disadvantaged communities, in view of seeking to improve health and care equity.

## Data

The dataset relied on in this article is available from the corresponding author on reasonable request.
